# Not part of my routine: a qualitative study of use and understanding of UV forecast information and the SunSmart app

**DOI:** 10.1186/s12889-019-7421-x

**Published:** 2019-08-16

**Authors:** Anna Nicholson, Michael Murphy, Heather Walker, Rick Tinker, Suzanne Dobbinson

**Affiliations:** 1 Centre for Behavioural Research in Cancer, Cancer Council Victoria, 615 St Kilda Road, Melbourne, Victoria 3004 Australia; 2 MMResearch, 9 School Lane, Ferny Creek, Victoria 3786 Australia; 3 SunSmart Victoria, Cancer Council Victoria, 615 St Kilda Road, Melbourne, Victoria 3004 Australia; 4 Australian Radiation Protection and Nuclear Safety Agency, 619 Lower Plenty Road, Yallambie, Victoria 3085 Australia

**Keywords:** Ultraviolet index, Sun protection, Skin cancer, Public education, Health promotion, Cancer prevention

## Abstract

**Background:**

The Ultraviolet (UV) Index provides a reliable means of monitoring the strength of UV radiation at the Earth’s surface, which can be used to indicate the potential for skin damage. This qualitative study aims to examine public understanding of the UV Index among Australians who routinely use UV forecast information as well as those who do not.

**Methods:**

Recent use of the SunSmart app (a popular mobile and tablet app that provides UV forecast information) served as a proxy for use of UV forecast information. Six focus groups were conducted with ‘new users’, who trialled the SunSmart app for the first time in the two weeks preceding the group discussion. In addition, 15 in-depth interviews were conducted with ‘existing users’, who routinely used the SunSmart app. Thematic discourse analysis was undertaken to compare views and experiences.

**Results:**

Misperceptions about UV radiation were common. Participants learnt new information about UV radiation, the UV Index, and the times of the day that sun protection is recommended. Among adults who were using UV forecast information for the first time, this learning rarely translated to new behaviours; for these participants, the UV Index forecast information and recommendations were inconsistent with their own observation of the weather and subsequent judgement about their propensity to burn. Thus new users considered the UV forecast information to be overly cautious. In contrast, existing users recognised their inability to judge the UV Index level; for these participants, UV forecast information provided by the SunSmart app was incorporated into their daily routines and used to inform their sun protection behaviours.

**Conclusions/implications:**

No matter how broadly UV forecast information is promoted, it is unlikely to improve sun protection behaviours across the Victorian population due to the low level of basic understanding of UV radiation. Public education strategies that correct the commonly held misperception that temperature and/or sunshine can reliably predict the potential for UV damage are required. Improved public awareness about UV radiation and how the UV Index can be used to prevent skin cancer may help Australians to develop more effective sun protection habits.

## Introduction

Australians have been taught to Slip Slop Slap since the 1980s [1]. We have made a lot of progress in this area nationally, with skin cancer rates now declining [2]. However, rapid improvements in sun protection and reduced sunburn in the first decade of the SunSmart program (1987–1997) have been followed with slower progress in the decade to 2007 [3]. Most skin cancers in Australia are preventable; high ambient levels of ultraviolet (UV) radiation are estimated to cause from 63 to 95% of melanomas and essentially all keratinocytic cancers [4, 5]. Skin cancers can be prevented by reducing harmful UV exposure and protecting the skin, including by the routine application of sunscreen [6].

Information about UV radiation is complex to communicate to the public, as UV Index levels vary with geographic location, season, altitude, cloud cover, ozone and other factors such as surface reflectance. It is the combination of these factors that determines how much UV radiation a person is exposed to direct from the sun, through scattering in the air and from surface reflection. The UV Index provides a reliable means of monitoring the strength of UV radiation at the Earth’s surface, which can be used to indicate the potential for skin damage. The World Health Organization (WHO) recommends use of sun protection whenever the UV Index reaches 3 and above [7]. In Australia, the duration of the day for which UV Index is forecast to reach 3 and above determines the UV forecast, or sun protection forecast (henceforce: the sun protection times). The Australian Bureau of Meteorology has been reporting the UV Index for over 20 years, and this information is routinely incorporated into a number of weather forecasting services. Despite that, routine use of the UV Index among members of the public is uncommon in Victoria and other States and Territories. The 2017 Bureau of Meteorology Public User Survey found that less than 1 in 2 Australian adults (47%) were aware that the Bureau reports the sun protection times and, of those aware, only 39% used the sun protection times to inform decisions about sun protection [8].

Although there is expert consensus regarding the usefulness of the UV Index as a skin cancer prevention tool [7], there is no evidence that awareness of the UV Index contributes to a reduction in skin cancer. A systematic review of UV forecast interventions found that information about that the UV Index was insufficient to prompt sun protective behaviours [9]. The review recommended more widespread dissemination of information about the UV Index that is accompanied by a clear call to action. Mobile phone interventions that deliver real-time UV information and sun protection advice relative to the user’s geographic location show potential to improve sun protection behaviours [10]. However, randomised controlled trials that test the effectiveness of UV radiation smartphone applications (apps) have not found evidence of reduced sunburn, due in part to low or inconsistent app use within the intervention group [11, 12]. It is also possible that persisting misperceptions of the UV Index may be undermining efforts to increase its use [13], including in the context of experimental research.

Launched in 2010, the SunSmart mobile and tablet app aims to communicate UV Index information in a way that is easy to understand, in order to influence decisions about sun protection. The SunSmart app communicates the daily sun protection times (i.e. when the UV Index is forecast to be 3 and above), which are accessible via the home page and an optional daily notification. Forecast information is provided by the Bureau of Meteorology. The SunSmart app also communicates the current UV Index level, which is updated in real-time for 13 locations around Australia, from a feed provided by the Australian Radiation Protection and Nuclear Safety Agency (ARPANSA). The SunSmart app has now been downloaded over 300,000 times. In 2016, Cancer Council Victoria partnered with ARPANSA to evaluate the effectiveness of the SunSmart app to communicate information about UV radiation and to inform decisions about sun protection. Due to a paucity of published research on public understanding of UV radiation, the study commenced with an exploration of public awareness of UV radiation and UV forecast information. This research was perceived to be critical to understanding findings regarding the effectiveness and impact of the SunSmart app.

This qualitative research paper aims to explore i) how Victorian adults use and understand information about UV radiation and ii) how the SunSmart app (a mobile and tablet app that provides UV forecast information and notifications) informs decisions about sun protection.

## Methods

Qualitative data were collected from six focus group discussions and fifteen interviews with adults resident in Victoria. Data were collected in November 2016. November presents an ideal context to explore understanding of the use of temperature and UV forecast information as, although the average UVI is high in Melbourne at that time of year (UVI maximum: 8) [14], anecdotal evidence suggests sun protection is less salient in spring, when the weather conditions are quite variable.

In order to represent the views and experiences of people who use UV forecast information as well as those who do not, participants were recruited and interviewed using two distinct sampling strategies and qualitative methods; focus groups were conducted with new users of the SunSmart app, and interviews were conducted with existing users of the SunSmart app. Here, use of the SunSmart app serves as a proxy measure for use of UV forecast information. This pragmatic approach enabled secondary data collection on specific features and functions of the SunSmart app (as part of an evaluation of the SunSmart app), which is not reported here.

A social research agency was engaged to recruit Victorian adults aged 18 to 44 years old to take part in one of six focus group discussions comprised of ‘new users’. Potential participants were excluded if they had downloaded and used the SunSmart app previously; if they reported their skin did not burn or tan following exposure to 30 min of strong sunshine in summer; or if they worked in the beauty, research or advertising industries. Groups were segregated according to the location of residence (Melbourne or Bendigo), age group (18–24, 25–34 or 35–44 years of age) and their parental status. Each group contained between 6 to 10 men and women. Focus group participants were requested to download and use the free SunSmart app in the two weeks prior to the group discussion. A qualitative researcher independent of SunSmart (MM) conducted all six focus group discussions to reduce the potential for SunSmart staff to bias the conversation. The discussion was facilitated according to a semi-structured interview guide, which allowed for probing of questions about understanding and use of UV forecast information and the SunSmart app. Each focus group discussion was audio-recorded and transcribed.

Interviewees (‘existing users’) were recruited using an online survey that was distributed via the SunSmart app. Adults who completed the survey and agreed to be contacted to take part in a telephone interview were included in a list of potential participants, which was stratified by sex, age group, household composition. The same social research agency recruited participants from each category in the stratified list. All 15 in-depth interviews were conducted by the same qualitative researcher as the focus group discussions (MM). Interviews were semi-structured and followed a similar schedule to the focus groups, with additional probing around drivers to download and use the SunSmart app in the first instance. Interviews took between 15 and 40 min and were audio-recorded and transcribed.

### Data analysis

Thematic discourse analysis was used to identify, analyse and report common viewpoints and experiences of participants in relation to: i) understanding about UV radiation and its effect on health; ii) use and interpretation of UV forecast information; iii) the influence of the SunSmart app on sun protection knowledge, intentions and behaviours. Semantic themes were identified by two analysts (MM and AN), who coded the data independently. Key themes were discussed and agreed upon by both analysts. Viewpoints and experiences were interpreted in the context of ‘new users’ (i.e. focus group participants) or repeat and regular ‘existing users’ (i.e. interviewees) of the SunSmart app.

## Results

Six focus groups (45 participants) and fifteen interviews (15 participants) were conducted in November 2016. Table 1 summarises group and participant characteristics, together with the notation used to attribute quotes through the results.

**Table 1:** Group and participant characteristics

Notation	Method	UV Index use	Group characteristics			Participants
			Age (years)	Residence	Parent	Gender
FG1	Focus group	New	25–34	Metro	No	Females: 4 Males: 4
FG2	Focus group	New	25–34	Metro	Yes	Females: 4 Males: 4
FG3	Focus group	New	18–25	Metro	No	Females: 4 Males: 4
FG4	Focus group	New	35–45	Metro	Yes	Females: 3 Males: 4
FG5	Focus group	New	18–24	Regional	No	Females: 4 Males: 3
FG6	Focus group	New	35–44	Regional	Yes	Females: 4 Males: 3
I1-I15	Interview	Existing: Use < 1 yr.: 5 Use ≥1 yr.:10	22–62	Mixed	Mixed	Females: 8 Males: 7

Key themes and quotes are summarised in three sections:

i) Understanding of information about UV radiation and its effect on health

ii) Use of UV forecast information

iii) Effectiveness of the SunSmart app to improve sun protection behaviours

In contrast to participants who had sought out UV forecast information, none of the participants who were prompted to download and trial the SunSmart app previously referred to UV forecast information. For this reason, there was very little familiarity of the UV Index or the sun protection times in any of the focus group discussions.

As viewpoints did not clearly differ by group characteristics, results have not been analysed by age, remoteness and parental status.

### Part 1. Understanding about UV radiation and its effects on health

#### Low levels of knowledge about UV radiation

There was consistent awareness that the sun is the source of UV radiation, that too much exposure to UV radiation can cause sunburn and skin cancer, and that not enough exposure to UV radiation can lead to vitamin D deficiency. Beyond that, focus groups and interviewees (together: ‘participants’) expressed limited understanding of UV radiation.

*It’s the heat from the sun, and if it’s too high that can be harmful, but … it depends on the level of the UV, because you need a certain amount to get your Vitamin D. (FG2, F)*

*Actually I know, UVB and UVA, so I know that there’s one that gives you colour and there’s another that gives you cancer. I don’t remember which one it is. (I4, F)*

*I know that if you don’t protect yourself from it, you end up with skin cancer...I don’t know all the scientific side of it. (I12, F)*

*I probably need to go back to school and learn about it or something. I don’t really understand it. (FG6, F)*

There was generally poor awareness of factors that influence UV Index levels. Three of the six groups identified the height of the sun in the sky as the main determinant of the UV Index level over the course of the day. Participants were also aware the UV Index levels vary between countries. However, it was common to attribute the hole in the ozone layer is the main cause of Australia’s high ambient UV Index levels and skin cancer rates. Further, some groups, particularly those with older participants, perceived that harm from UV radiation had increased due to problems associated with the ozone layer and/or climate change.

*Australia is in a place that’s not protected by the ozone layer as much as other countries so we’re more susceptible to burning. (FG3, F)*

*I think of like climate change, in relation to like the ozone layer … So the need for this sort of promotion is probably a lot higher now, than what it would have been. (FG5, F)*

#### Myths and misconceptions

Misconceptions about UV radiation were common among focus group participants, who had not previously used UV forecast information routinely. Many groups expressed uncertainty at UV Index levels and associated harm on cool and overcast days. Sunny weather and clear skies were a more sensitive trigger for sun protection than changing seasons, which led to daily variation in sun protection behaviours.

*When I think of burns I think of the sun hitting me. But when I’m behind a cloud there’s no sun hitting you. (FG3, M)*

*I think theoretically I knew that clouds didn’t mean there wasn’t extreme UV but I just always associated sun’s out I could get sunburnt. If it’s not then I’d be alright. (FG4, M)*

For some participants, experiencing sunburn in unexpected conditions, including on cool or cloudy days, when sitting in shade, and at the snow, had challenged pre-conceptions about UV radiation.

*I’ve managed to get myself sunburned on an overcast day. ..Yeah, but if that didn’t happen to me, I will definitely be in the camp of ‘oh it’s not warm, why?’ (FG5, M)*

*The problem with cloud cover and temperature, the colder and cloudier it is, the more you perceive that it’s not as bad, but you know, I’ve been out once or twice where it’s been really, really cloudy and I’ve been burned. So I think it’s kind of like learn by mistake. (I10, M)*

However, sunburn on overcast days was commonly mislabelled as windburn.

*I would never get burnt in winter. Though like unless it’s like super windy and you get wind burn or something … (FG3, F)*

*FG6, F1: There seems to be a lot more windburn as well. Do you notice that?*

*FG6, F2: Yeah, I get that.*

*FG6, F1: I don’t really get the windburn thing …*

*FG3, F3: I was going say, is that a real thing or is it just sunburn disguised, because we think it’s windburn because it was cloudy? I don’t know.*

#### Conflicting advice on how to prevent skin cancer and maintain vitamin D

Participants expressed confusion about how to best maintain Vitamin D levels, which was deepened by conflicting advice, including from medical professionals. A small number of groups perceived that too much emphasis had been placed on skin cancer prevention at the expense of maintaining adequate vitamin D levels.

*I keep getting conflicting information all the time. Even from doctors. (I8, F)*

*Now everyone is lacking vitamin D and they’re all taking tablets because they’ve slipped, slopped, slapped so much they haven’t got any exposure. (FG1, M)*

*In the 80’s and the 90’s there was obviously huge incidence of skin cancer, and then from there, there was this big Slip Slop Slap campaign that came out in the 90’s, and then we had like this, over the next fifteen years, a big push of Vitamin D deficiency across Australia as well. And that was happening when I was in university, and I thought well okay, maybe we should be a little bit more relaxed with it. So, it’s very confusing. (FG2, M)*

### Part 2. Use of UV forecast information

#### Interpretation of the UV index

When asked about the UV Index, focus group participants were unable to describe or interpret the scale.

*I’d like the number to have some relevance because I’m not educated on the UV factor and how it pertains to skin danger and stuff … .(FG4, M)*

*I didn’t really need to know what the numbers were, that really wasn’t a priority to me. If it’s telling me when I’m in danger it really doesn’t matter what the numbers are … (I12, F)*

Whilst the sun protection times were “intuitive”, they were also considered to be too prescriptive and overly cautious. For example, high and very high UV ratings were considered the most appropriate threshold for sun protection (rather than moderate).

*To be truthful I’d probably only put sunscreen on if it was over 7 or 8 maybe. (FG2, M)*

*And because it’s moderate, you think it’s not too bad. But as soon as it says high, that’s my threshold. (FG1, F)*

#### Perceived value of UV forecast information

Due to familiarity with the Slip Slop Slap message, sun protection was framed as “common sense” (FG2, F). For many, looking out the window to see whether the sun is out was deemed to be sufficient to guide their decisions about sun protection. As such, not only were the sun protection times considered unnecessary, the lack of day to day variation (as for sunshine) fuelled mistrust in this information.

*I was a bit dubious as to how much I should really believe it, because it felt like it never really tailored to what I was seeing, outside the window. (FG2, F)*

*Yeah, so even though it’s telling me that I should, wear sun protection, if I don’t feel like it’s that hot, that sunny I don’t really worry about it too much. (FG5, F)*

In contrast, existing users of UV forecast information discussed the utility of this information to prevent skin cancer, which they were motivated to address either personally or for others in their care.

*I had got a melanoma myself, so I use it now just to check UV so I know when to put sunscreen on. (I8, F)*

*I’ve obviously downloaded this app, because it was something that I was personally already fairly committed to and invested in. (I7, F)*

Existing users, who had sought out UV forecast information via the SunSmart app independent of this study, recognised that their own judgement of the weather conditions was insufficient to prevent harmful UV exposure.

*It may look overcast but the UV is still high and I couldn’t judge that, so that’s why I was looking for something that would just tell me, because I’m not really good at judging these things. (I12, F)*

*… there are kind of assumptions which I’ve always made about, like what times of the year and what kind of weather correlates with a high UV Index. And those assumptions are pretty much wrong. (I13, M)*

The stark contrast in the perceived value and utility of UV forecast information had important implications for whether and how it was applied.

*This is probably not the right thing to say but I still feel a level of apathy towards it. I don’t really care. I just know that if the sun’s out I need to wear sunscreen and I don’t really care about the science behind it, to be honest. (FG1, F)*

### Part 3. Effectiveness of the SunSmart app to improve sun protection behaviours

#### New learning

Both existing and new users of the SunSmart app reported learning about UV radiation through their use of the SunSmart app. Repeat observations of UV radiation patterns throughout the day (and over time) helped to shift misconceptions about UV radiation.

*I guess some of the stuff on it was a shock to begin with and I realised I didn’t really know that much, about the UV side of things. (I8, F)*

*If I didn’t know about that app I wouldn’t have known about the UV … it’s like a big realisation for me. (FG3, F)*

*Something else I picked up was like it was cold outside or cool, but the UV was actually still quite high. (FG5, F)*

The recommendation to protect the skin from UV Index 3 and above was also new information to almost all participants, as was the duration of the day the UV Index levels are 3 and above in spring in Victoria.

*So, it’s helped me learn … essentially about three quarters of the year I should be using sunscreen (I13, M)*

*I wasn’t aware that you needed to have sunscreen on if it was over 3. That seemed quite a low level. (FG2, M)*

*Because my mum always said from 11 until 3 stay under the tree … and then on the app it’s until 5 o’clock, so it’s a lot longer than I would’ve thought. (FG1, F)*

#### Acceptance of new information

Most groups expressed surprise and scepticism toward the sun protection times. The duration of the day that sun protection is recommended during spring in Victoria was longer than expected and inconsistent with prior learning. For these reasons, some saw the sun protection times as an overprotective recommendation.

*Overprotective. This coming from a pale person as well. (FG1, M)*

*They’re just being cautious, it’s kind of like a disclaimer. (FG1, F)*

*I kind of thought that if you follow that, you’d be going nuts putting on sunscreen every time you stepped out of the door. But I don’t know. (FG2, F)*

For participants who doubted the forecast information, their beliefs were often justified by their understanding of their skin type and of how much sun they can tolerate before burning. As most participants used sun protection in order to avoid sunburn (rather than to prevent cumulative harm or skin cancer), the instruction to use sun protection from UV Index 3 and above was dismissed as overly cautious.

*I don’t know, maybe 3, 4, 5 people get away with it, whereas 10 or 11, you’re going to burn. (FG1, F)*

*I always thought that you get those sort of problems if you have serious burns over and over again, or like you are really into tanning (FG2, M)*

*It would be good to know exactly what the risks are. The feeling I get is that 3, 4, 5 the damage can’t be that bad … (FG2, F)*

However parents were not willing to take the same risk for their children:

*I think from my perspective I probably* *would* *question it. When I think about my son … I don’t want to expose him to anything … I’ve got to do the right thing and make sure he’s all covered up (FG1, F)*

*If I did have kids I think I would 100%. But if it’s me I’m not too worried. (FG4, M)*

*And I also think a 4 for me is different to a 4, for my 1 year old. So I’d probably be more inclined to put sunscreen on them if it was a 3, 4, 5 than myself … (FG2, F)*

#### Influence on sun protection behaviours and routines

Despite the new learning reported within each focus group, this did not always translate to behaviour. Some groups discussed thinking about the need for sun protection more than usual, but not acting upon this:

*I’ve thought about using it more, but … (FG5, F)*

*I’m more aware of it but my behaviour hasn’t changed. (FG3, M)*

*It’s like today I was out on a walk with my dog and I was like, I probably should’ve put sunscreen on, because I the app reminded me … but I didn’t … (FG3, F)*

One group of older adults reflected the reason they didn’t act upon this advice was *not* because it wasn’t perceived to be important, but that it wasn’t at the forefront of their mind.

*I don’t deliberately not think about it. It’s just not part of my routine, it’s not part of what I do. (FG6, F)*

In other groups, participants lacked well-established sun protection habits reflected that the SunSmart app helped to make sun protection front of mind. For a small number of participants, the daily notification about the sun protection times had triggered new behaviours that had the potential to develop into new routines.

*I set it [the notification] for my lunch time. And it’s actually prompted me, okay, how long am I going to spend outside in my lunch break, and do I need to put a layer of sunscreen on before I go out. (FG2, F)*

*It’s part of life, the brushing teeth routine now, it’s just a part of it. And that’s being directly related to this [app]. (FG4, F)*

In contrast, almost all interviewees (who had sought out the SunSmart app independent of this research) reported that sun protection had been incorporated into their daily routines; to prepare for the day, to avoid peak UV radiation periods, and to plan time outdoors for young children.

*Every day, it gives me a reminder over day of when’s the time to have the sunscreen on (I4, F)*

*… every day I check before I leave the house. So that I’m sort of organised for my day, just like knowing whether I need to bring a jumper, I know whether I need to put on sunscreen on. (I14, F)*

Even though the SunSmart app and current UV Index levels were checked less frequently with time, the daily notifications helped to keep sun protection front-of-mind, irrespective of other weather conditions and competing demands.

*I think the other good thing it does is just remind you all the time, not that you need a lot of reminding with blue skies and baking heat but, just keeps it front of mind, particularly at the times when maybe the risk is not quite as obvious. (I3, M)*

*Because of the morning notifications, I know each day, like every single day, whether I need it or not, and it sort of gives me a bit of a reminder, Like it plants a seed in the back of my mind, just like: ‘I should use sunscreen today’. (I13, M)*

## Discussion

Among participants who did not routinely use UV forecast information, the degree of sunshine on any given day was perceived to be sufficient to guide decisions about sun protection. Whether UV forecast information was seen to be valuable was largely determined by whether people felt confident to judge their risk of sunburn for the day. Participants’ poor understanding of the relationship between UV Index levels and other weather factors was contributing to them getting ‘caught out’, resulting in unexpected sunburn on cool and cloudy days. Further, as the desire to avoid sunburn was the main driver of sun protection, most groups saw little reason to routinely protect the skin from UV Index of 3 and above in order to prevent damage from cumulative UV exposure. Only those who were driven to protect their skin in order to avoid prevent skin cancer (rather than sunburn) accepted and incorporated UV forecast information into their sun protection habits and routines.

We found low levels of understanding about the UV radiation and the UV Index, which contributes to low awareness and use of UV forecast information [8]. As such, the status quo of making UV forecast information available for those who seek it is likely insufficient to prevent UV damage at a population level. Although widespread and easily accessible UV forecast information will increase exposure to UV information in the general public [9], this research suggests that may not be sufficient to increase use of this information. Consistent with previous qualitative research, [13] common misperceptions about UV radiation continue to undermine the use of UV forecast information to develop sun protection habits and routines. In particular, we found a clear need to correct the misperception that temperature and sunshine are useful indicators of the potential for skin damage. Similarly, it is important to communicate that not all skin damage is visible in order to shift the public focus on avoiding sunburn to one of preventing cumulative damage from UV radiation.

A mass media campaign that tackles common myths about UV radiation could go some way to improve public understanding of when sun protection is required. Although there are no published evaluations of mass media campaigns that focus on UV myth-busting, there is strong evidence that well-televised skin cancer prevention advertisements reduce pro-tanning attitudes and skin exposure and increase sunscreen use among adolescents and adults [15, 16]. Although public education is not its primary objective, the SunSmart app also shows promise as a tool to educate Australians about daily and seasonal variations in UV radiation (and UV Index levels); which could be valuable to teachers, health professionals and other skin cancer prevention educators. However, as isolated attempts to raise awareness of the UV Index are unlikely to improve sun protection behaviours [9], a comprehensive approach that integrates education about UV radiation with other skin cancer prevention strategies is required. For example, combining lessons for secondary students with strategies that tackle sun protection norms and the school environment is likely to be more effective than providing skin cancer prevention education in isolation [17]. Public education is just one of many skin cancer prevention strategies that contribute to Australia’s comprehensive and cost-effective skin cancer prevention efforts [18–20].

This study summarises the views and experiences of a small number of Victorians. By including adults who did not routinely use UV forecast information as well as those who did (via the SunSmart app), we heard from adults with various levels of interest in the topic. Although we cannot be sure that more viewpoints would have arisen had we continued the research, common and repeat themes were evident at the conclusion of the focus groups and interviews. It is a clear limitation of the research that we did not speak with people from other States and Territories; however that was not feasible within the scope of work. The varying climates across Australia are likely to introduce regional differences in barriers to using UV forecast information to inform sun protection behaviours. It would be valuable to extend this work to other areas of Australia, including more northerly and tropical regions, where sun protection is recommended year-round, and in regions that have aired public education campaigns about the UV Index.

This research was conducted as part of an evaluation of the SunSmart app. As such, the SunSmart app was the only UV forecast modality that was made available for road-testing and feedback. It is possible that participation in the study prompted participants to access other sources of information about UV radiation prior to the group discussion. However, it is the intention for this article to focus on insights regarding the *application* of learning about UV forecast information, rather than the UV Index display or mode of delivery. Our research offers some explanation for why the UV Index has been found to have limited effect on motivating sun protection behaviours; however it does not consider whether the UV Index is an effective tool to communicate information about UV radiation. Further research that considers whether understanding about UV radiation mediates appropriate use of the UV Index to inform sun protection behaviour would be a useful addition to this field.

## Conclusions

This exploratory study provides valuable insight to how Victorians understand and use UV forecast information. Tools such as the SunSmart app have potential to improve understanding about UV radiation and to inform sun protection routines among those who seek it out. However, no matter how broadly it is promoted, UV forecast information is unlikely to improve sun protection behaviours across the Victorian population due to the low level of basic understanding about UV radiation. Public education strategies that correct the commonly held misperception that temperature and/or sunshine can reliably predict the potential for UV damage are required. Improved public awareness of UV radiation and how the UV Index can be used to prevent skin cancer may go some way to helping Australians develop more effective sun protection habits as part of a comprehensive approach to skin cancer prevention.

## Data Availability

The data that support the findings of this study are not publically available due to privacy and confidentiality restrictions. Requests to access the data can be made to the corresponding author (AN), however access will be limited by the conditions under which participant consent was granted.

## Abbreviations

ARPANSA: Australian Radiation Protection and Nuclear Safety Agency
UV: Ultraviolet
UVI: Ultraviolet Index
